# Extended-Spectrum β-Lactamase-Producing *Enterobacterales* Shedding by Dogs and Cats Hospitalized in an Emergency and Critical Care Department of a Veterinary Teaching Hospital

**DOI:** 10.3390/antibiotics9090545

**Published:** 2020-08-27

**Authors:** Anat Shnaiderman-Torban, Shiri Navon-Venezia, Efrat Kelmer, Adar Cohen, Yossi Paitan, Haya Arielly, Amir Steinman

**Affiliations:** 1 Koret School of Veterinary Medicine (KSVM), The Robert H. Smith Faculty of Agriculture, Food and Environment, The Hebrew University of Jerusalem, Rehovot 7610001, Israel; ashnaiderman@gmail.com (A.S.-T.); kelmere1@gmail.com (E.K.); adarkohen@gmail.com (A.C.); 2 Department of Molecular Biology, Faculty of Natural Science, Ariel University, Ariel 40700, Israel; shirinv@ariel.ac.il; 3 The Miriam and Sheldon Adelson School of Medicine, Ariel University, Ariel 40700, Israel; 4 Department of Clinical Microbiology and Immunology, Sackler Faculty of Medicine, Tel Aviv University, Tel Aviv 6997801, Israel; yossi.paitan@clalit.org.il; 5 Clinical Microbiology Lab, Meir Medical Center, Kfar Saba 4428164, Israel; Ariellyhaya@clalit.org.il

**Keywords:** ESBL-PE, antibiotic resistance, companion animals, emergency and critical care

## Abstract

Extended-spectrum β-lactamase-producing *Enterobacterales* (ESBL-PE) gut shedding in human medicine is considered as a major reservoir for ESBL-associated infections in high risk patients. In veterinary medicine, data regarding ESBL-PE gut shedding on admission to emergency and critical care department is scarce. We aimed to determine ESBL-PE shedding rates by dogs and cats in this setting and to determine the risk factors for shedding, at two separate periods, three-years apart. Rectal swabs were collected from animals, on admission and 72 h post admission, enriched and plated on Chromagar ESBL plates, followed by bacterial identification. ESBL phenotype was confirmed and antibiotic susceptibility profiles were determined (Vitek 2). Medical records were reviewed for risk factor analysis (SPSS). Overall, 248 animals were sampled, including 108 animals on period I (2015–2016) and 140 animals on period II (2019). In both periods combined, 21.4% of animals shed ESBL-PE on admission, and shedding rates increased significantly during hospitalization (53.7%, *p*-value < 0.001). The main ESBL-PE species were *Escherichia coli* and *Klebsiella pneumoniae*, accounting for more than 85% of the isolates. In a multivariable analysis, previous hospitalization was a risk factor for ESBL-PE gut shedding (*p*-value = 0.01, Odds ratio = 3.05, 95% Confidence interval 1.28–7.27). Our findings demonstrate significant ESBL-PE gut shedding among small animals in the emergency and critical care department, posing the necessity to design and implement control measures to prevent transmission and optimize antibiotic therapy in this setting.

## 1. Introduction

Extended-spectrum β-lactamases (ESBL) enzymes enable bacteria to hydrolyze penicillins, cephalosporins and monobactams, thus conferring resistance which is limiting the therapeutic options [1]. ESBL producing *Enterobacterales* (ESBL-PE) colonize various body sites, such as the intestinal and urinary tract, and may cause infections in these body systems, as well as pneumonia and bloodstream infections [2]. In human medicine, ESBL-PE gut shedding by patients is considered as a major reservoir for ESBL-associated infections both in the community and in hospitals [3]. Furthermore, according to several studies in human hospitals, ESBL-PE gut colonization increases the risk of a subsequent ESBL-PE infection in high-risk patients [4,5]. This was recently demonstrated in a study from Switzerland, where ESBL-PE colonization on admission to the intensive care unit in the University Hospital Basel was associated with subsequent ESBL-PE infection [6]. The suggested pathomechanism is transition of colonizing bacteria from the impaired intestinal tract to the bloodstream [2], which highlights the importance of ESBL-PE colonized patients not only as reservoirs but also as high-risk patients for developing infections. Therefore, on hospital admission, sampling is essential for both identification of patients at risk for developing ESBL-PE infection and for the prevention of ESBL-PE spread among other high-risk patients [7].

In the recent years, several studies described ESBL-PE colonization and infection in dogs and cats [8,9]. Infections caused by ESBL-PE in dogs and cats include abscesses and wounds, otitis, upper respiratory tracts diseases, gastro-intestinal infections and cystitis [8,10]. Colonization was also described worldwide, with rates ranging from 6% to 24% in different geographical regions and different cohorts [11,12,13,14]. Recent studies described co-carriage of ESBL producing *Escherichia coli (E. coli)* and *Klebsiella pneumoniae (K. pneumoniae)* strains in humans and dogs of the same household [15,16]. These findings highlight the importance of investigating shedding rates and risk factors for shedding by dogs and cats in both veterinary and ‘one health’ perspectives.

Several studies investigated ESBL-PE gut shedding and infection rates in healthy and in hospitalized dogs and cats [17], but data regarding shedding rates and risk factors of ESBL-PE on admission to the emergency and critical care department is scarce. This data is crucial to understand the epidemiology of ESBL-PE shedding in emergency veterinary medicine, in specific in an emergency and critical care department setup, in which patients are in life-threatening situations that require intensive medical treatments. Understanding ESBL-PE gut shedding in this cohort is essential to design control measures and prevent the environmental spread of ESBL-PE, and for the identification of animals at high risk for ESBL-PE infection.

In this study, we aimed to determine the ESBL-PE gut shedding rates in dogs and cats admitted to the emergency and critical care department, at two different periods, three-years apart. The analyses included identification of the ESBL-PE bacterial species, their antibiotic susceptibility patterns, and the risk factors for shedding on admission and during hospitalization in this department.

## 2. Results

### 2.1. Population Characteristics

**Figure 1:**
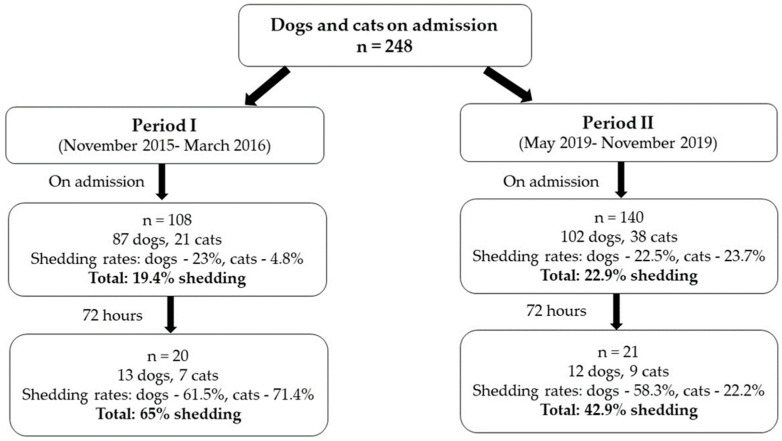
Gut shedding of ESBL-PE in dogs and cats admitted to the small animal emergency and critical care department during two periods-study design.

Shedding of ESBL-PE in dogs and cats admitted to the small animal emergency and critical care department was studied during two periods. During period I, 108 patients were sampled on admission and 20 patients were re-sampled 72 h post admission (Figure 1). Of those animals that were sampled on admission, 87 were dogs, which belonged to 33 different breeds, and 21 were cats, all belonged to one breed. The most common cause of admission was having a gastrointestinal disease (31.7%), 28.9% of the animals were treated with antibiotics within the previous year and 13% were hospitalized in the previous year, with a median hospitalization length of two days (Supplementary Table S1).

During Period II, 140 patients were sampled and 21 patients were re-sampled 72 h post admission (Figure 1). Of those animals that were sampled on admission, 102 were dogs, which belonged to 34 different breeds, and 38 were cats, which belonged to eight different breeds. The most common cause of admission was having a gastrointestinal disease (24.1%), similarly to period I. Antibiotic treatment within the previous year was documented in 28.4% of animals, and 20.9% of animals were hospitalized in the previous year. The median hospitalization length was three days (Supplemental Table S1).

Overall, 28.6% of all the sampled animals were treated with antibiotics within a year prior to admission to the department (Supplementary Table S1). The most prevalent antibiotic therapy was β-lactams, excluding carbapenems that were not used at all (Table 1). The population characteristics in both periods (I and II) was almost similar. The only significant difference was that previous admission to a veterinary clinic was higher (*p* = 0.008) during period II (Supplementary Table S1).

### 2.2. ESBL-PE Gut Shedding Rates

Data on the ESBL-PE gut shedding rates in animals during the two study periods is presented in Table 2. Overall, for both periods combined, the ESBL-PE gut shedding rates increased during hospitalization (72 h post admission), from 21.4% to 53.7% (*p* < 0.001).

In order to determine the acquisition and the persistence of ESBL-PE during hospitalization in the emergency and critical care department, we re-sampled all animals that were still hospitalized 72 h after admission (41 animals in both periods). Of these that were non-ESBL-PE carriers on admission (*n* = 27), 59.3% remained negative and 40.7% acquired ESBL-PE (*de novo* shedders) during hospitalization; of the ESBL-PE on admission shedders (*n* = 14), 71.4% remained positive and 28.6% turned negative during hospitalization. The total acquisition rate of ESBL-PE during hospitalization was 26.8% (11/41, nine animals in period I and two animals in period II).

### 2.3. Distribution of the ESBL-PE Bacterial Species

#### 2.3.1. On Admission 

**Figure 2:**
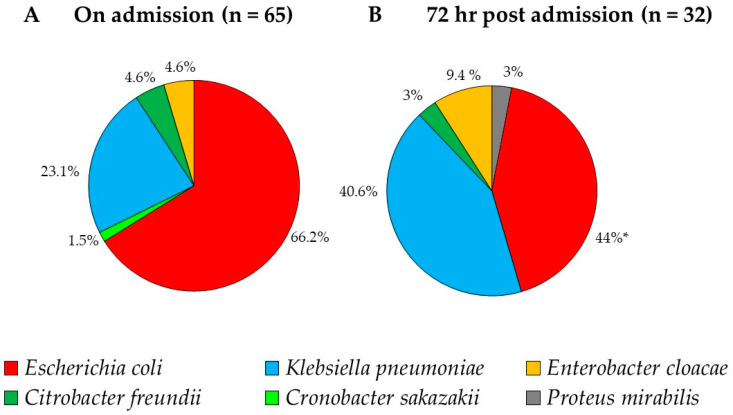
Bacterial species isolated from dogs and cats on admission (I, *n* = 65) and 72 h post admission (II, *n* = 33) to the small animal emergency and critical care department. * A significant decrease in *E. coli* prevalence (*p* = 0.048).

On admission, during period I, 26 ESBL-PE isolates were recovered belonging to three bacterial species with *E. coli* being the major species–69.2%, following with *K. pneumoniae*–23.1% and *Citrobacter freundii*–7.7%. During Period II, 39 bacteria were isolated, including five bacterial species: 64.1% *E. coli,* 23.1% *K. pneumonia,* 7.7% *Enterobacter cloacae,* 2.55% *Cronobacter sakazakii* and 2.6% *Citrobacter freundii*. The relative prevalence of the ESBL-PE species on admission was similar between periods I and II, therefore we present the overall prevalence combining period I and II. The most prevalent species on admission were *E. coli* (66.2%, 43/65, 95% CI 53.4–77.4) and *K. pneumoniae* (23.1%, 15/65, 95% CI 13.5–35.2) (Figure 2A).

#### 2.3.2. During Hospitalization 

During hospitalization (72 h post admission), during period I, 19 bacterial isolates were isolated, including five bacterial species: 52.6% *E. coli,* 26.3% *K. pneumoniae*, 10.5% *Enterobacter cloacae*, 5.3% *Citrobacter freundii* and 5.3% *Proteus mirabilis.* During Period II, 13 bacterial isolates were isolated, including three bacterial species: 61.5% *K. pneumoniae*, 30.8% *E. coli* and 7.7% *Enterobacter cloacae.* There were no statistical differences in the prevalence of the bacterial species between period I and II. Overall, combining periods I and II, the most prevalent bacterial species during hospitalization were *E. coli* and *K. pneumoniae,* accounting for 84.6% of all the isolates. We noticed a significant decrease in *E. coli* prevalence during hospitalization (*p* = 0.048), compared to on admission, and no significant change in the prevalence of the other bacterial species. The increase in *K. pneumoniae* prevalence (1.8-fold) was insignificant (*p* = 0.096).

Data describing the ESBL-PE species recovered from hospitalized animals, some of which acquired more than one species, and their susceptibility patterns are summarized (Supplementary Table S2). Of 41 animals that were re-sampled at 72 h, 24.4% (*n* = 10/41) acquired *K. pneumoniae*, 24.4% (*n* = 10/41) acquired *E. coli*, two animals (a cat and a dog) acquired *Enterobacter cloacae*, and single animals acquired *Proteus mirabilis* and *Citrobacter freundii*. *Escherichia coli* was persistent in three dogs (7.3%, 3/41, 95% CI 15.4–19.2) and *K. pneumoniae* was persistent in one dog (2.4%, 1/41, 95% CI 0.6–15.4) (Figure 2B).

### 2.4. Susceptibility Patterns of the ESBL-PE Isolates

During period I, there was a significant decrease in resistance rates to amoxicillin-clavulanate and a significant increase in resistance rates to ofloxacin (*p* < 0.05, Table 3). During period II, there was a significant increase in resistance rates to ofloxacin and nitrofurantoin (*p* < 0.05).

Comparing resistance rates to different antibiotics between period I and II on hospital admission, there was a significant decrease in resistant rates to amoxicillin-clavulanate and a significant increase in resistance rates to nitrofurantoin (*p* < 0.05, Table 3). Other resistance rates between the periods were not significantly different, on both admission and 72 h post admission (Table 3).

There was a significant increase in multi-drug resistance rates between admission and hospitalization on period II (Table 3). All bacterial isolates were susceptible to imipenem.

### 2.5. Risk Factor Analysis for ESBL-PE Gut Shedding

#### 2.5.1. Period I

In a univariable analysis of dogs, the following categorical variables were associated with ESBL-PE gut shedding on admission: hepatic disease and respiratory disease (*p* < 0.05, Table 4). These variables, as well as cardiovascular disease were analyzed in a logistic regression model and were found to be non-significant (*p* > 0.05, Table 5). In a univariable analysis of cats, no variables were significantly associated with gut shedding. In a univariable analysis of dogs and cats together, hepatic disease, respiratory disease and the animal species were included in a logistic regression model (*p* < 0.2, Table 4). Respiratory disease was identified as the only risk factor for ESBL-PE gut shedding on admission (Table 5).

#### 2.5.2. Period II

In a univariable analysis of dogs, the following variables were associated with ESBL-PE gut shedding on admission: hematologic disease, respiratory disease and weight (*p* < 0.05, Table 4). These variables, as well as amoxicillin-clavulanate treatment, were analyzed in a logistic regression model. The variable “weight” was identified as a risk factor for ESBL-PE gut shedding (Table 5). In a univariable analysis of cats, admission to a veterinary clinic in the previous year, hospital admission in the previous year and weight were associated with ESBL-PE gut shedding on admission (*p* < 0.05, Table 4. These variables were non-significant in a logistic regression model (*p* > 0.05, Table 5).

In a univariable analysis of dogs and cats together, ESBL-PE gut shedding on admission was associated with shedding 72 h post admission, previous hospital admission, respiratory disease and weight (*p* < 0.05, Table 4). These variables (excluding shedding 72 h post admission), as well as admission to a veterinary clinic in the previous year, injury, and a hematological disease were analyzed in a logistic regression model. Weight was identified as a risk factor for shedding on admission (Table 5).

#### 2.5.3. Periods I and II

In a univariable analysis of dogs, weight was significantly associated with ESBL-PE gut shedding on admission (*p* < 0.05, Table 4). In a univariable analysis of cats, hospital admission in the previous year was significantly associated with ESBL-PE gut shedding on admission (Table 4). In a logistic regression model, including also weight, admission to a veterinary clinic in the previous year and injury, none of these variables were identified as risk factors for ESBL-PE gut shedding (Table 5).

In a univariable analysis of dogs and cats together, ESBL-PE gut shedding on admission was significantly associated with animal weight and with hospital admission in the previous year (*p* < 0.05, Table 3). These variables, as well as injury, were analyzed in a logistic regression model. Hospital admission in the previous year was identified as a risk factor for ESBL-PE gut shedding on admission (Table 5).

## 3. Discussion

This study investigated the prevalence and risk factors for ESBL-PE gut shedding by dogs and cats on admission to an emergency and critical care department in a veterinary teaching hospital, during two periods. To our knowledge, this study is unique as it focusses specifically on shedding of antibiotic resistant ESBL-PE and defines risk factors for gut shedding in this population. Understanding the burden of ESBL-PE shedding in complicated animal patients in the hospital vicinity is highly valuable for designing control measures. Minimizing resistance spread and the identification of patients at risk for developing ESBL-PE infection is still understudied in veterinary medicine.

Overall, during the two periods, we screened 248 dogs and cats on admission. The veterinary referral center studied here is the largest emergency center in the country and treats animals from all over the country. Data regarding dogs and cats was collected and analyses were performed in several perspectives—(i) the two time periods, (ii) dogs versus cats and (iii) combined analyses—taking into consideration both animal species and the two-time periods. Although we investigated two different animal species, we chose to examine the combined data of dogs and cats in addition to a separate species analyses, due to similar housing conditions in the community, similar animal-human contact and similar hospitalization conditions.

The overall ESBL-PE shedding rate on admission was 21.4% and was insignificantly different between the periods. Shedding rate on admission does not necessarily represent the healthy companion animal community, since these animals were referred to a tertiary referral center, 63.2% of them were previously treated in a veterinary clinic and 17.7% were hospitalized in the previous year. This shedding rate is similar to what was found among horses in the same veterinary hospital, where ESBL-PE shedding upon hospital admission was 19.6% [18]. Data from human medicine reported in Israel more than a decade ago, indicated lower ESBL-PE shedding rates upon hospital admission of 13.7% [19] and 10.7% in another study [20]. The comparison to human population is important in the perspective of ‘one health’, since companion animals live in close contact to humans, but these studies were performed in different periods and in dissimilar set-ups, and therefore, further studies are warranted for a reliable comparison.

Analyzing the data during the two periods revealed similarities between the two periods with respect to the population characteristics and the ESBL-PE shedding rates, both on admission and 72 h post admission (Supplementary Table S1). Although the two periods are three-years apart, we did not find an increase in the prevalence of ESBL-PE gut shedding (Supplementary Table S1), nor in the antibiotic usage (Table 1). In a recent European study, investigating the antimicrobial usage and resistance in companion animals, 19% of animals received at least one antimicrobial treatment six months preceding sampling, with the most frequently used antimicrobial was amoxicillin-clavulanate [21]. In our study, 28.6% of animals were treated one-year preceding sampling, and β-lactams, excluding carbapenems were the most frequently used group. The comparable result may imply similar antibiotic stewardships in companion animals’ medicine in the recent years.

During hospitalization, the ESBL-PE gut shedding rate increased significantly to 53.7%, overall ESBL-PE acquisition rate was 26.8%, *E. coli* acquisition rate was 24.4% and persistent in 7.3% of the animals. These findings could be the result of acquisition of resistant bacteria or mobile genetic elements from the hospital environment. Alternatively, these could be the result of an increase in resistant bacteria that were undetected in the gastrointestinal flora as was previously suggested [18,22]. In a similar study in a veterinary teaching hospital in the United States, multidrug resistant (MDR) *E. coli* was acquired in 6.8% of the animals and was persistent in 3% [22]. However, data on ESBL-producing *E. coli* was not examined in this later study. These different trends are interesting findings that could have been driven by a number of factors, including different population characteristics, antibiotic stewardships and variation in the study design. Despite the arising numbers of different studies regarding ESBL-PE shedding and infections in a variety of companion animals’ cohorts, there is a lack of evidence regarding the association between ESBL-PE shedding and infections. In human medicine, ESBL-PE gut shedding has been identified as a risk factor for infection [4,6] and this should be further studied in animals as well.

The main ESBL-PE species were *E. coli* and *K. pneumoniae*, as previously reported in companion animals [13,23,24]. There was no significant change in species prevalence between on-admission and during hospitalization, in both periods and between periods. The only significant difference was the decrease in *E. coli* prevalence post admission, when combining both periods, alongside with insignificant increase in *K. pneumoniae* prevalence. In similar studies conducted in the large animal department in the same veterinary teaching hospital and during similar time periods, the main ESBL-PE species found were *E. coli* and *K. pneumoniae*, but there were significant changes in bacterial species distribution, including new species that were acquired during hospitalization [18,25]. This may be due to differences in pathologies and antibiotic stewardships between the small and large animals as well as differences in hospitalization facilities, and therefore calls for further investigation.

We found differences in bacterial antibiotic resistance patterns between periods I and II (Table 3). In ESBL-PE isolates obtained 72 h post admission during period II we found a significant increase MDR and specifically in resistance rates to ofloxacin and nitrofurantoin. Nitrofurantoin is not widely used in veterinary medicine owing to its pharmacokinetics and adverse clinical effects [26]. However, nitrofurantoin is commonly used for treatment of uncomplicated urinary tract infection in otherwise healthy young women [27]. Co-selection of resistance to aminoglycosides, quinolones and tetracycline is prevalent among ESBL-producers as was previously described in human ESBL isolates and in environmental samples [28,29,30]. Fluoroquinolones are frequently used in veterinary medicine [31], and therefore further research is needed to ascertain the gravity of quinolones resistance and the overall MDR among nosocomial veterinary pathogens.

In human medicine, several studies were conducted on carriage of ESBL-PE on admission to emergency and intensive care units. Shedding rates varied dramatically between reports in different countries from 4 to 62.5% [6,32,33] and risk factors for ESBL-PE shedding included elderly age, cirrhosis, broad-spectrum antibiotic treatment, urinary or intra-abdominal infections and residence in overcrowded households districts [34,35]. Hospitalization in the previous year was identified as a risk factor for ESBL-PE shedding in dogs and cats in both periods (*p* =0.01, OR = 3.05, 95% CI 1.28–7.27, Table 5), as reported before in human medicine [20,36]. This is an important finding and should be considered in decision making for implementing active surveillance in veterinary clinics. The duration of ESBL-PE carriage was beyond the scope of this study. In human medicine, ESBL-PE shedding duration varies significantly between different populations, from 59 days to over one year [37,38,39]. A longitudinal study on ESBL-PE carriage in healthy dogs presented duration of at least six months [40], but data regarding duration following hospital discharge is lacking. Additional studies are required in order to predict suspected ESBL-PE shedding animals on admission.

The limitations of this study include a small sample size collected in each period, small number of cats and a retrospective medical data collection. Even though the statistical analysis revealed significant risk factors, a larger sample size may have resulted in the identification of additional risk factors that could differentiate between ESBL-PE gut shedding among dogs and cats. Unfortunately, data regarding ESBL-PE infection in these animals was not available, therefore conclusions regarding the association between ESBL-PE colonization and infection could not be drown. Another limitation is that we only selected one colony from each of the colors/morphology for our analysis, which may result in missing information about other clones. This study emphasizes the importance of applying an active surveillance policy for ESBL-PE shedding in small animals admitted to emergency and critical care department in veterinary hospitals. Future studies should include a larger cohort and further investigate the association between ESBL-PE shedding and infections caused by these antibiotic resistant pathogens.

## 4. Materials and Methods

### 4.1. Study Design

We conducted a case-control study in the small animal emergency and critical care department in the Koret School of Veterinary Medicine-Veterinary Teaching Hospital (KSVM-VTH), during two time periods, three years apart: period I (November 2015–March 2016) and period II (May 2019–November 2019). During period I, 108 patients (87 dogs and 21 cats) were sampled on admission and 20 patients (13 dogs and 7 cats) were re-sampled 72 h post admission. During period II, 140 patients (102 dogs and 38 cats) were sampled on admission and 21 patients (12 dogs and 9 cats) were re-sampled 72 h post admission. All animals that survived and were not discharged were re-sampled 72 h post-admission. The study was approved by the Internal Research Committee of the KSVM, Israel (Protocol KSVM-VTH/15_2015). Sampling of all animals required owners’ approval and was performed on admission prior to any medical treatment or procedure in the hospital.

### 4.2. Isolation of ESBL-PE Gut Shedding and Species Identification

Rectal specimens were collected using bacteriological swabs (Meus s.r.l., Piove di Sacco, Italy) and were inoculated directly into a Luria Bertani infusion enrichment broth (Hy-Labs, Rehovot, Israel) to increase the sensitivity of ESBL-PE detection [17]. After incubation at 37 °C (18–24 h), enriched samples were plated onto Chromagar ESBL plates (Hy-Labs, Rehovot, Israel), at 37 °C for 24 h. Colonies that appeared after overnight incubation at 37 °C were recorded, and one colony of each distinct color and/or morphology was re-streaked onto a fresh Chromagar ESBL plate to obtain a pure culture. Pure isolates were stored at −80 °C for further analysis.

Isolates were subjected to Vitek-MS (BioMérieux, Inc., Marcy-l’Etoile, France) for species identification or to Vitek-2 (BioMérieux, Inc., Marcy-l’Etoile, France) for species identification and/or antibiotic susceptibility testing (AST-N270 Vitek-2 card). Species identification by Vitek-MS was performed according to the manufacturer instructions. Briefly, isolated colony was sampled onto the MS slid followed by addition of 1µL VITEK MS CHCA, the slide was inserted, after drying, into the Vitek-MS for identification. Positive identification after spectrum analysis with Confidence Level above 95 was considered as good identification. Species identification and/or antibiotic susceptibility testing by Vitek-2 using the VITEK 2 GN card for identification and the AST-N270 card for susceptibility testing according to the manufacturer instructions. In addition, susceptibility of ofloxacin and imipenem were analyzed using disc diffusion assay (Oxoid, Basingstoke, UK) according to the Clinical and Laboratory Standards Institute (CLSI) guidelines [41,42]. ESBL production was tested and confirmed with the CLSI confirmatory test using both CTX (30 mg) and CAZ (30 mg) (Oxoid, Basingstoke, UK) disks alone and in combination with CA (10 mg) (Sensi-Discs BD, Breda, The Netherlands). The test was considered positive when an increase in the growth-inhibitory zone around either the CTX or the CAZ disk with CA was 5 mm or greater of the diameter around the disk containing CTX or CAZ alone. Results were interpreted according to the CLSI guidelines [41,42]. Isolates were defined MDR based on an in vitro resistance to three or more classes of antimicrobial agents [43]. A nosocomial ESBL-PE acquisition was defined when an animal became an ESBL-PE shedder during hospitalization, or when a new ESBL-PE species was isolated at 72 h post admission compared to admission. A persistent ESBL-PE species was defined when the same animal shed this species on admission and at 72 h post admission.

### 4.3. Demographic and Medical Data

Medical records were reviewed for the following information: signalment (species, age, sex and breed); weight; admission to any veterinary clinic within the previous year (yes/no); admission to the hospital within the previous year (yes/no); clinical signs on admission; duration of illness before admission; antibiotic therapy within a year prior to hospitalization (yes/no and also divided by antibiotic classes); hospitalization length of stay and short-term outcome.

### 4.4. Statistical and Risk Factor Analyses

Statistical analyses were performed using the IBM STATISTICS SPSS software (SPSS Version 24; SPSS Inc, Chicago, IL, USA). Data distribution was examined by testing whether the Skewness and kurtosis equal zero and by performing the Shapiro-Wilk’s test. Continuous variables were analyzed using t-tests or Mann-Whitney U-tests, according to the distribution of the variable. Categorical variables were analyzed using the Fisher’s exact test or the Pearson chi-square test, as appropriate. In all statistical analyses, *p* ≤ 0.05 indicated significance. A multiple logistic regression model, using the ENTER method, was applied for ESBL-PE shedding using variables with *p* ≤ 0.2 [44]. Due to the retrospective design, prevalence and rate were calculated as valid percentage, whereas missing data was removed from mechanism. Rates and confidence intervals were calculates using the WinPepi software (version 11.62) [45].

## 5. Conclusions

This study substantiates the significance of ESBL-PE shedding by dogs and cats on admission to an emergency and critical care department, and during hospitalization. Further studies and active surveillance should focus on community-onset, nosocomial ESBL-PE shedding and association with ESBL-PE infection.

## Figures and Tables

**Table 1:** Previous antibiotic treatments in dogs and cats prior to admission to the small animal emergency and critical care department.

Period	Animal		Antibiotic Therapy Within A Year Prior To Admission (% Valid Percentage) ^1^				
		Penicillins ^2^	Amoxicillin-Clavulanate	Cephalosporins ^3^	Quinolones	Doxycycline	Metronidazole
I	Dogs (*n* = 87)	13.5	4.1	4.1	4.1	1.4	4.2
	Cats (*n* = 21)	13.3	13.3	0	13.3	0	13.3
	Total (*n* = 108)	13.5	5.7	3.4	5.7	1.1	5.7
II	Dogs (*n* = 102)	4.9	8.5	3.7	3.7	6.1	3.7
	Cats (*n* = 38)	0	9.7	6.5	0	3.2	0
	Total (*n* = 140)	3.5	8.8	4.5	2.7	5.3	2.7
I & II	Dogs (*n* = 189)	9	6.9	3.9	3.9	3.9	3.9
	Cats (*n* = 59)	4.3	11.9	4.3	4.3	2.2	4.3
	Total (*n* = 248)	8	7.6	4.1	4	3.5	4.1

^1^ Valid percent-missing data was removed from the denominator. ^2^ Including: amoxicillin, ampicillin and penicillin. ^3^ Including: cefazolin and cefalexin.

**Table 2:** ESBL-PE gut shedding rates on admission to small animal emergency and critical care department and following 72 h of hospitalization.

Period	Animal	ESBL Gut Shedding Rate		
		on Admission % (Frequency, 95% CI)	At 72 h % (Frequency, 95% CI)	*p*-Value ^1^
I	Dogs (*n* = 87)	23 (20/87, 14.6–33.3)	61.5 (8/13, 31.6–86.1)	0.007 *
	Cats (*n* = 21)	4.8 (1/21, 0.1–23.8)	71.4 (5/7, 29–96.3)	0.001 *
	Total (*n* = 108)	19.4 (21/108, 12.5–28.2)	65 (13/20, 40.8–84.6)	<0.001 *
II	Dogs (*n* = 102)	22.5(23/102, 14.9–31.9)	58.3 (7/12, 27.7–84.8)	0.014 *
	Cats (*n* = 38)	23.7 (9/38, 11.4–40.2)	22.2 (2/9, 2.8–60)	1
	Total (*n* = 140)	22.9 (32/140, 16.2–30.7)	42.9 (9/21, 21.8–69.0)	0.062
I & II	Dogs (*n* = 189)	22.8 (43/189, 17–29.4)	60 (15/25, 39.7–78.9)	<0.001 *
	Cats (*n* = 59)	16.9 (10/59, 8.4, 29)	43.8 (7/16, 19.8–70.1)	0.04 *
	Total (*n* = 248)	21.4 (*n* = 53/248, 16.4–27.0)	53.7 ^1^ (*n* = 22/41, 37.4–69.3)	<0.001 *

^1^ A comparison between ESBL-PE gut shedding rate on admission and at 72 h post admission, in the same raw. All other comparisons, between the same animal species in different periods or between cats and dogs on the same period- were not significantly different. * *p* < 0.05.

**Table 3:** Antibiotic resistance rates of ESBL-PE isolates originated from dogs and cats hospitalized in an emergency and critical care department in two periods.

Period	Sampling (Number of Isolates)	AMC (95% CI)	OFL (95% CI)	AMK (95% CI)	GEN (95% CI)	TMS (95% CI)	NIT (95% CI)	MDR (95% CI)
Period I	Admission (*n* = 26)	100 (86.7–100)	44 (24.4–65.1)	3.8 (0.1–19.6)	15.4 (4.4–34.9)	65.4 (44.3–82.8)	7.7 (0.9–25.13)	69.2 (48.2–85.7)
	72 h post admission (*n* = 19)	69.2 ^1^ (38.6–90.9)	87.5 ^3^ (61.7–98.5)	0 (0–17.7)	41.2 (18.4–67.1)	88.2 (63.6–98.5)	5.9 (0.2–28.7)	94.4 (72.7–99.9)
Period II	Admission (*n* = 39)	28.2 ^2^ (15–44.9)	53.9 (37.2–69.9)	0 (0–9)	38.5 (23.4–55.4)	71.8 (55.1–85)	17.95 (7.5–33.5)	61.5 (44.6–77.6)
	72 h post admission (*n* = 13)	46.2 (19.2–74.9)	100 ^4^ (73.5–100)	0 (0–24.7)	38.5 (13.9–68.4)	84.6 (54.5–98.1)	53.85 ^5,6^ (25.2–80.8)	92.3 ^7^ (64–99.8)

^1^ A significant decrease in resistance rate to AMC (amoxicillin-clavulanate) during period I (admission versus 72 h post admission), *p* < 0.001. ^2^ A significant decrease in resistance rate to AMC between period I and II, on admission, *p* < 0.001. ^3^ A significant increase in resistance rate to OFL (ofloxacin) during period I, *p* = 0.008. ^4^ A significant increase in resistance rate to OFL during period II, *p* = 0.004. ^5^ A significant increase in resistance rate to NIT (nitrofurantoin) during period II, *p* = 0.026. ^6^ A significant increase in resistance rate to NIT between period I and II, 72 h post admission, *p* = 0.009. ^7^ A significant increase in multidrug resistance rate 72 h post admission, on period II, *p* = 0.044.

**Table 4:** Univariable analyses for ESBL-PE gut shedding on hospital admission to the small animal emergency and critical care department.

Period		Period I			Period II			Period I & II		
Variables (*p*-Value)		Dogs	Cats	Dogs & Cats	Dogs	Cats	Dogs & Cats	Dogs	Cats	Dogs & Cats
Demographics	Species ^1^			0.07 ^6^			0.89			0.38
	Gender ^2^	0.52	0.41	0.42	0.29	0.41	0.7	0.87	0.23	0.98
	Breed	0.82	1	0.62	0.63	0.64	0.62	0.75	0.78	0.77
	Age	0.87	NI ^5^	0.94	0.36	0.27	0.74	0.44	0.23	0.81
	Weight	0.8	0.57	0.45	0.01 *^,6^	0.08	0.03 *^,6^	0.04 *^,6^	0.19 ^6^	0.048 *^,6^
Medical background	Previous admission to a veterinary clinic ^3^	0.26	1	0.22	1	0.03 *^,6^	0.16 ^6^	0.52	0.17 ^6^	0.89
	Previous hospitalization ^3^	1	1	1	0.24	0.03 *^,6^	0.02 *^,6^	0.313	0.02 *^,6^	0.035 *^,6^
	Length of illness before admission	0.4	NI	0.35	0.88	0.77	0.93	0.66	0.77	0.49
Previous antibiotic treatments ^3^	Antibiotic treatment (yes/no)	0.73	0.4	0.54	0.92	1	0.74	0.85	1	0.92
	Penicillins ^4^	0.66	0.13	0.37	1	NI	1	1	0.28	0.51
	Amoxicillin-clavulanate	0.42	0.13	0.16	0.19 ^6^	1	0.12 ^6^	0.69	1	0.74
	Cephalosporines	1	NI	1	1	1	0.59	0.6	1	0.36
	Quinolones	0.42	0.13	0.16	0.55	NI	0.53	0.35	0.28	0.18
	Doxycycline	1	NI	1	0.33	1	0.61	0.35	1	0.62
	Metronidazole	0.43	1	1	0.55	NI	0.53	0.35	1	0.652
Clinical syndrome on admission	Neurological disease	0.73	1	1	0.56	0.63	0.4	0.4	1	0.45
	Injury	1	1	1	0.45	0.31	0.12 ^6^	0.57	0.19 ^6^	0.11 ^6^
	Cardiovascular disease	0.07 ^6^	1	0.21	1	0.66	0.76	0.22	0.67	0.58
	Hematologic disease	0.68	0.053	1	0.028 *^,6^	1	0.14 ^6^	0.25	0.51	0.27
	Gastro-intestinal disease	0.54	1	0.59	0.49	1	0.46	0.37	1	0.4
	Endocrinopathy	NI	NI	NI	1	1	0.57	1	1	0.58
	Hepatic disease	0.046 *^,6^	1	0.09 ^6^	0.57	1	0.68	0.62	1	0.72
	Reproduction related disease	1	NI	1	0.59	NI	0.59	0.2	NI	0.21
	Respiratory	0.02 *^,6^	1	0.055 ^6^	0.04 *^,6^	1	0.04 *^,6^	0.82	0.67	0.8
	Orthopedic	0.68	1	1	1	0.56	0.3	0.53	0.58	0.26
	Intoxication	1	NI	1	0.41	1	0.54	0.66	1	0.68
	Ophthalmological	1	1	1	0.57	0.22	1	0.6	0.3	1
	Tumor	0.29	NI	0.46	0.59	0.56	0.95	0.62	1	0.55
	Urinary-tract disease	1	1	0.73	1	0.37	0.56	0.8	0.42	0.94
Outcomes	Hospital discharge (yes/no)	0.73	0.08	0.75	0.76	0.37	0.8	0.55	1	0.78
	ESBL-PE gut shedding 72 h post admission	1	1	1	0.24	0.17	0.03 *	0.23	0.6	1
	Length of stay	0.48	0.56	0.45	0.14 ^7^	0.9	0.23	0.44	1	0.55
	Length of stay excluding dead	0.52	^8^	0.27	0.28	0.57	0.57			0.96

^1^ Only for dogs and cat analyses. ^2^ Four categories: intact male, intact female, castrated male and spayed female. ^3^ During the previous year. ^4^ Including amoxicillin and amoxicillin-clavulanate. ^5^ NI—not identified, there is not enough data for analysis. ^6^ Included in a multivariable analysis due to *p* ≤ 0.2. ^7^ “Length of stay” was not included in a multivariable analysis, since this is an outcome and not a risk factor. ^8^ The distribution of this variable is the same across categories of ESBL-PE gut shedding at admission, therefore this test cannot be computed. * *p* ≤ 0.05.

**Table 5:** Logistic regression analyses for ESBL-PE gut shedding on hospital admission to the small animal emergency and critical care department.

Period	Period I		Period II			Periods I & II	
Variable (*p*-Value, OR, 95% CI)	Dogs	Dogs & Cats	Dogs	Cats	Dogs & Cats	Cats	Dogs & Cats
Species ^1^	NI ^3^	0.09 OR = 0.16 0.02–1.35	NI	NI	NI	NI	NI
Previous admission to a veterinary clinic ^2^	NI	NI	NI	0.999	0.14 OR = 0.19 95% CI 0.03–1.47	0.774 OR = 0.7 95% CI 0.6–7.6	NI
Previous hospital admission ^2^	NI	NI	NI	0.92 OR = 0.76 0.002–232	0.095 OR = 5.82 1.28–7.27	0.56 OR = 0.7 95% CI 0.6–7.6	0.01 * **OR = 3.05** **1.28–7.27**
Amoxicillin-clavulanate before admission ^2^	NI	NI	>0.99	NI	0.999	NI	NI
Injury on admission	NI	NI	NI	NI	0.999	0.999	0.4 OR = 0.51 0.11–2.4
Cardiovascular disease on admission	0.42 OR = 2.05 0.36–11.74	NI	NI	NI	NI	NI	NI
Hematologic disease on admission	NI	NI	0.12 OR = 4.35 0.68–27.84	NI	0.35 OR = 2.54 0.36–17.86	NI	NI
Hepatic disease on admission	1	0.069	NI	NI	NI	NI	NI
Respiratory disease on admission	0.08 OR = 3.43 0.87–13.49	0.029 * **OR = 3.63** **1.15–11.5**	>0.99	0.99	0.998	NI	NI
Weight (Kg)	NI	NI	0.014 * **OR = 1.07** **1.01–1.13**	0.07 OR = 3.69 0.89–15.26	0.011 * **OR = 1.1** **1.02–1.19**	0.3 OR = 1.33 0.78–2.25	0.07 OR = 1.02 0.99–1.05

^1^ Only for dogs & cats analyses. ^2^ During the previous year. ^3^ NI—Not Included due to *p* > 0.2 in the univariable analysis (Table 4). * *p* < 0.05.

## Supplementary Materials

The following are available online at https://www.mdpi.com/2079-6382/9/9/545/s1, Table S1: Characterization of dogs and cats admitted to the emergency and critical care department. Table S2: ESBL-PE species recovered from hospitalized animals sampled on admission and 72 h post admission to the small animal emergency and critical care department.
