# Supplementary material for: Extended-Spectrum β-Lactamase-Producing Enterobacterales Shedding by Dogs and Cats Hospitalized in an Emergency and Critical Care Department of a Veterinary Teaching Hospital
Source: Antibiotics (Basel). 2020 Aug 27;9(9):545. doi: 10.3390/antibiotics9090545 (PMC7557403; doi:10.3390/antibiotics9090545)
Supplement: Supplementary file 1 [file antibiotics-09-00545-s001.zip › Supp Table S1.docx]

**Supplemental Table S1. Characterization of dogs and cats admitted to the emergency and critical care department**

| **Period** | **Animals** | **Gender**  **(valid %)** | **Main breed**  **(valid %)** | **Median Age (years) (range)** | **Weight**  **(Kg)**  **(range if median)**  **±SD if mean** | **Previous**  **Admission to a veterinary clinic ^1^**  **(valid %)** | **Previous Hospital admission ^1^ (valid %)** | **Previous antibiotics treatment ^2^ (valid %)** | **Main pathologies on admission ^2, 3^**  **(valid %)** | **Median Illness length before admission**  **(days) (range)** | **Median LOS**  **(days) (range)** | **Median LOS after excluding dead (days) (range)** | **Short term outcome- hospital discharge**  **(valid %)** |
| --- | --- | --- | --- | --- | --- | --- | --- | --- | --- | --- | --- | --- | --- |
| **I** | Dogs (n=87) | Intact female: 14.8 Spayed: 33.3  Intact male: 32.1 Castrated: 19.8 | Mixed: 46.3 | 9  (0.25- 17) | 20.5  (2.5- 84.6) | 50.7 | 14.7 | 26.7 | Gastro-intestinal (GI) : 32.9  Neurological & Respiratory: 18.3 each | 2 (0-150) | 2 (0-8) | 2 (0-8) | 77.9 |
|  | Cats (n=21) | Spayed: 42.1  Intact male: 15.8 Castrated: 42.1 | Mixed: 100 | 5  (0.16- 14) | 4.16± 0.45 | 58.8 | 5.9 | 40 | Neurologic: 26.6  Respiratory & GI : 26.3 each | 1.5 (1-14) | 2.5 (1-10) | 3 (1-10) | 72.2 |
|  | Total (n=108) | Not relevant ^4^ | Not relevant ^4^ | Not relevant ^4^ | Not relevant ^4^ | 52.3 | 13 | 28.9 | GI: 31.7  Neurology: 19.8  Neurological & Respiratory: 18.5 each | 2 (0-150) | 2 (0-10) | 2 (0-10) | 76.8 |
| **II** | Dogs (n=102) | Intact female: 8.9 Spayed: 34.7  Intact male: 34.7 Castrated: 20.8 | Mixed: 42.6 | 8 (0.08-16) | 17.84±13.94 | 72.6 | 21.6 | 31 | GI: 25  Tumor: 22  Neurology: 21 | 4 (0-270) | 3 (1-18) | 2.5 (1-18) | 81.2 |
|  | Cats (n=38) | Intact female: 13.2 Spayed: 28.9  Intact male: 5.3 Castrated: 52.6 | Mixed: 60.5 | 4.75 (1-17) | 4.1 (1.93-10.5) | 67.7 | 18.9 | 21.9 | Urinary: 24.3  GI & Cardiovascular: 21.6 | 2 (0-120) | 2 (0-16) | 2 (0-16) | 73 |
|  | Total (n=140) | Not relevant ^4^ | Not relevant ^4^ | Not relevant ^4^ | Not relevant ^4^ | 71.3 | 20.9 | 28.4 | GI: 24.1  Tumor and urinary: 19  Respiratory: 15 | 3 (0-270) | 3 (0-18) | 2 (0-18) | 79 |
| **I & II** | Dogs  (n=189) | Intact female: 11.5 Spayed: 34.6  Intact male: 33.5 Castrated: 20.3 | Mixed: 42.3 | 8 (0.08-17) | 17 (2.3-84.6) | 62.7 | 18.6 | 28.9 | GI: 28.6  Tumor: 19.8  Respiratory & urinary: 15.9 | 3 (0-270) | 2 (0-18) | 2 (0-18) | 79.9 |
|  | Cats  (n=59) | Intact female: 8.8 Spayed: 33.3  Intact male: 8.8 Castrated: 49.1 | Mixed: 69.5 | 4.75  (0.16-17) | 4 (0.94-10.5) | 64.6 | 14.8 | 27.7 | GI and urinary: 23.2  Respiratory & Neurology: 21.4 | 2 (0-120) | 2 (0-16) | 3 (0-16) | 72.7 |
|  | Total  (n=248) | Not relevant ^4^ | Not relevant ^4^ | Not relevant ^4^ | Not relevant ^4^ | 63.2 ^5^ | 17.7 | 28.6 | GI: 27.3  Respiratory: 17.2 Neurology: 20.2 | 3 (0-270) | 2 (0-18) | 2 (0-18) | 78.2 |

^1^ Valid percent - missing data was removed from the denominator.

^2^ During the previous year before admission.

^3^ Some animals presented more than one clinical sign.

^4^ Combination of dogs and cats data is not relevant in these categories, due to species differences.

^5^ Significantly higher on period II compared to period I (*P*=0.008).
