# Supplementary material for: Extended-Spectrum β-Lactamase-Producing Enterobacterales Shedding by Dogs and Cats Hospitalized in an Emergency and Critical Care Department of a Veterinary Teaching Hospital
Source: Antibiotics (Basel). 2020 Aug 27;9(9):545. doi: 10.3390/antibiotics9090545 (PMC7557403; doi:10.3390/antibiotics9090545)
Supplement: Supplementary file 1 [file antibiotics-09-00545-s001.zip › Supp Table S2.docx]

**Supplementary Table S2. ESBL-PE species recovered from hospitalized animals sampled on admission and 72 hours post admission to the small animal emergency and critical care department**

|  |  |  | **On admission** | |  | **72 hours post admission** |
| --- | --- | --- | --- | --- | --- | --- |
| **ID** | **Animal species** | **Period** | **Bacterial**  **Species** | **Resistance**  **phenotype** | **Bacterial**  **Species** | **Resistance**  **phenotype** |
| **36** | Dog | I | *E. coli* | AMC-OFL-TMS | None | None |
| **41** | Dog | I | None | None | *E. coli* | AMC-GEN-NIT-TMS |
|  |  |  |  |  | *Proteus mirabilis* | GEN-NIT-TMS |
| **50** | Cat | I | None | None | None | None |
| **51** | Cat | I | None | None | *E. coli* | TMS |
| **52** | Dog | I | None | None | *K. pneumoniae* | AMC-OFL- TMS |
| **61** | Cat | I | None | None | *E. coli* | Not preserved for analysis |
|  |  |  |  |  | *Citrobacter freundii* | AMC-OFL-GEN-TMS |
| **71** | Cat | I | *K. pneumoniae* | AMC-OFL-TMS | *E. coli* | AMC-OFL-GEN |
| **73** | Dog | I | *E. coli* | AMC-OFL-AMK-GEN-TMS | *E. coli* | AMC-OFL-GEN-TMS |
|  |  |  | *Citrobacter freundii* | AMC |  |  |
| **86** | Dog | I | None | None | *E. coli* | OFL-NIT-TMS |
|  |  |  |  |  | *K. pneumoniae* | AMC- OFL-NIT-TMS |
| **100** | Dog | I | *K. pneumoniae* | AMC-NIT-TMS | *K. pneumoniae* | AMC-OFL-GEN-NIT-TMS |
|  |  |  |  |  | *E. coli* | AMC-OFL-GEN- NIT-TMS |
| **104** | Cat | I | None | None | *E. coli* | OFL- TMS |
| **119** | Dog | I | None | None | *Enterobacter cloacae* | AMC-OFL-GEN-NIT-TMS |
| **129** | Dog | I | None | None | *Klebsiella pneumoniae* | AMC-NIT-TMS |
| **184** | Dog | I | None | None | None | None |
| **186** | Dog | I | None | None | *K. pneumoniae* | AMC-OFL-NIT-TMS |
| **199** | Cat | I | None | None | *K. pneumoniae* | AMC-OFL-NIT-TMS |
| **255** | Dog | I | None | None | None | None |
| **257** | Dog | I | None | None | None | None |
| **258** | Dog | I | None | None | None | None |
| **285** | Cat | I | None | None | None | None |
| **A14** | Dog | II | *E. coli* | AMC-OFL | *E. coli* | AMC-OFL |
|  |  |  |  |  | *K. pneumoniae* | AMC-OFL-NIT-TMS |
| **A15** | Dog | II | *E. coli* | OFL-GEN-TMS | *E. coli* | AMC-OFL-NIT-TMS |
| **A19** | Cat | II | None | None | None | None |
| **A24** | Dog | II | None | None | None | None |
| **A27** | Cat | II | *E. coli* | OFL-TMS | *K. pneumoniae* | AMC-OFL-NIT-TMS |
| **A30** | Dog | II | *E. coli* | AMC | *K. pneumoniae* | AMC-OFL-GEN-NIT-TMS |
| **A53** | Dog | II | None | None | None | None |
| **A68** | Cat | II | None | None | None | None |
| **A71** | Dog | II | *Enterobacter cloacae* | AMC | *E. coli* | OFL-NIT |
| **A74** | Dog | II | *E. coli* | Susceptible for  AMC-OFL-AMK-GEN-NIT-TMS | *K. pneumoniae* | AMC-OFL-GEN-NIT-TMS |
| **A102** | Cat | II | *E. coli* | OFL-NIT | None | None |
| **A108** | Dog | II | None | None | None | None |
| **A112** | Dog | II | None | None | *E. coli* | AMC-OFL-TMS |
| **A117** | Cat | II | None | None | None | None |
| **A118** | Dog | II | None | None | None | None |
| **A119** | Cat | II | *E. coli* | TMS | *K. pneumoniae* | AMC-OFL-GEN-NIT-TMS |
|  |  |  |  |  | *Enterobacter cloacae* | AMC-OFL-GEN-NIT-TMS |
| **A120** | Dog | II | *K. pneumoniae* | NIT-TMS | None | None |
| **A122** | Dog | II | None | None | *E. coli* | OFL-TMS |
|  |  |  |  |  | *K. pneumoniae* | OFL-TMS |
| **A128** | Cat | II | *E. coli* | Susceptible for  AMC-OFL-AMK-GEN-NIT-TMS | None | None |
| **A142** | Cat | II | None | None | None | None |
| **A147** | Cat | II | None | None | None | None |
